# Development of a Core Outcome Measurement Set for studies of community pharmacist-led medication reviews in older patients with multimorbidity and polypharmacy

**DOI:** 10.1007/s11096-026-02223-y

**Published:** 2026-09-16

**Authors:** Cathrin J. Vogt, Christiane Eickhoff, Veerle Foulon, Carmel Hughes, Jens Kessler, Cinara Paul, Cristín Ryan, Martin Schulz, Martina Teichert, Ronja Woltersdorf, Viktoria S. Wurmbach, Hanna M. Seidling

**Affiliations:** 1 Heidelberg University, Medical Faculty Heidelberg / Heidelberg University Hospital, Internal Medicine IX – Department of Clinical Pharmacology and Pharmacoepidemiology, Cooperation Unit Clinical Pharmacy, Im Neuenheimer Feld 410, 69120 Heidelberg, Germany; 2 Department of Medicine, ABDA-Federal Union of German Associations of Pharmacists, Berlin, Germany; 3 Department of Pharmaceutical and Pharmacological Sciences, KU Leuven, Louvain, Belgium; 4 School of Pharmacy, Queen’s University Belfast, Belfast, Northern Ireland; 5 Medical Faculty Heidelberg, Department of Anesthesiology, Pain Medicine Section, Heidelberg University, Heidelberg, Germany; 6 Department of General Internal Medicine and Psychosomatics, University Hospital Heidelberg, Heidelberg, Germany; 7 Institute for General Practice and Interprofessional Care, Tübingen University Hospital, Tübingen, Germany; 8 School of Pharmacy and Pharmaceutical Sciences, Trinity College Dublin, Dublin, Republic of Ireland; 9 Institute of Pharmacy, Freie Universität Berlin, Berlin, Germany; 10 Primary Health Care, Leiden University Medical Center, Leiden, The Netherlands; 11 Department of Clinical Pharmacy, Institute of Pharmacy, University of Bonn, Bonn, Germany

**Keywords:** Community pharmacy, Core outcome set, Core outcome measurement set, Instruments, Medication review, Operationalization

## Abstract

**Introduction:**

A Core Outcome Set (COS) can harmonize outcome reporting. For medication review (MR) studies targeting older, multi-morbid patients with polypharmacy, a COS with seven outcomes is available: Health-related Quality of Life (HRQoL), pain relief, overuse, underuse, potentially inappropriate medications (PIMs), clinically significant drug-drug-interactions (DDIs), drug-related hospitalization. However, the lack of standardized outcome measurement is a barrier to its application. Thus, the next step is to operationalize each outcome by standardizing their measurement, resulting in a Core Outcome Measurement Set (COMS). As operationalization is setting-specific, a COMS was developed for MR studies in community pharmacies. Depending on the outcome type, the aims were to (1) identify outcome measurement instruments (OMIs), (2) define quality criteria for OMI selection, (3) refine outcome definitions, and/or (4) seek agreement on the appropriate measurement methods.

**Method:**

Literature searches were conducted to identify OMIs. A group of 10 experts was assembled, with at least one expert contributing to each outcome. Throughout the study, they completed two online surveys and took part in two online workshops. Based on the type of outcome, different approaches were undertaken: (1) The experts selected the most appropriate OMIs for HRQoL and pain relief. (2) Recognizing national differences in OMIs for overuse, underuse and PIMs, experts established universal quality criteria for OMIs’ selection to be applied for studies. (3) To operationalize DDIs and drug-related hospitalization, experts refined the definitions of what to measure. (4) For drug-related hospitalization experts sought to identify the best measurement method.

**Results:**

(1) The experts selected the EQ-5D-5L as most appropriate OMI for HRQoL and upon numerical and visual analogue scales for pain relief. (2) Seven quality criteria were defined for the content of OMIs on overuse, underuse, and PIMs. No thresholds were established for validity and feasibility criteria. (3) DDIs to be measured were refined as those with a 'major' severity rating. Drug-related hospitalization was refined as unplanned overnight stays; (4) measured by patients’ reporting.

**Conclusion:**

By translating the existing COS into a practical measurement framework, this COMS supports more standardized and comparable MR research.

**Trial registration:**

Registered on 16 August 2023 at Open Science Framework (https://osf.io/57jfc).

**Supplementary Information:**

The online version contains supplementary material available at https://doi.org/10.1007/s11096-026-02223-y.

## Impact statements

- This study operationalizes the existing Core Outcome Set (COS) for medication review studies by translating predefined outcomes into clear and actionable guidance for their measurement.
- It introduces a Core Outcome Measurement Set (COMS) specifically developed for community pharmacist-led medication review studies.
- By offering structured and practical guidance on outcome measurement, this COMS will enhance consistency and strengthen the robustness of evidence in future medication review research.

## Introduction

Medication reviews (MRs) aim to improve appropriate medication use [1, 2], and community pharmacists are well positioned to deliver them due to their expertise and accessibility [3–5]. However, evidence on the effectiveness of MRs remains often inconclusive due to heterogeneous outcome reporting [6, 7]. To harmonize outcome reporting, the development of Core Outcome Sets (COS) is promoted, i.e., a standardized set of outcomes that should be measured for a specific condition or intervention [8]. COS can be developed for research or clinical practice and are agreed upon by health professionals, researchers, policymakers, and patients or their representatives [9]. Despite increasing availability, COS uptake remains low [10–12]. Barriers include lack of awareness, difficulty identifying appropriate COS and researchers' preferences for selected outcomes [12–15]. Another prominent challenge is the uncertainty about which instruments or methods are appropriate to measure these outcomes [12, 16]. While a COS defines ‘what’ to report, it is also necessary to establish ‘how’ the outcomes should be measured. The Outcome Measures in Rheumatology (OMERACT) and COnsensus-based Standards for selection of health Measurement INstruments (COSMIN) initiatives provide guidance on selecting outcome measurement instruments (OMIs). It is recommended that OMIs are identified through systematic reviews or other sources, their methodological quality in the target setting and population is assessed, and only one OMI per core outcome is defined by stakeholder consensus [17, 18]. The resulting set of standardized OMIs is called a Core Outcome Measurement Set (COMS).

A COS for MR studies in multi-morbid older patients with polypharmacy has been available since 2018 [19]. It includes two patient-reported outcomes (Health-Related Quality of Life (HRQoL), pain relief), four medication-use outcomes (overuse, underuse, potentially inappropriate medications (PIMs), clinically significant drug-drug interactions (DDIs)); and one adverse-event outcome: drug-related hospitalization.

## Aim

However, uptake of the MR COS has been low [6], likely due to limited guidance on how to measure these outcomes. Thus, the aim of this work was to develop a COMS specifically for community pharmacist-led MR studies involving older patients with multimorbidity and polypharmacy. Depending on the type of outcome, different objectives were pursued. For well-defined outcomes, such as HRQoL and pain relief, the aim was to identify the most appropriate OMIs. In contrast, the assessment of overuse, underuse, and PIMs is highly context- and country-specific; therefore, the aim was to establish universal quality criteria to support the selection of appropriate OMIs. For DDIs and drug-related hospitalization, the objective was to refine outcome definitions to improve conceptual clarity and operationalization. Finally, an appropriate method for measuring drug-related hospitalization was sought.

## Method

### Formation of an expert group

This study used a multiphase, evidence-informed expert panel approach. Experts were purposively sampled to ensure representation across all core outcomes and were selected based on their clinical, research, or methodological expertise in MRs, implementation science, questionnaire development, or outcome measurement. Potential participants were identified through the study team’s professional networks, relevant scientific publications, and the Pharmaceutical Care Network Europe (PCNE) Symposium 2024 in Basel, where the project was presented and attendees invited to participate. All participants provided written informed consent.

Two rounds of 90-min online workshops and two accompanying surveys (Supplementary file 1) were conducted. The first workshop was held in September 2024 across two sessions to accommodate participants’ availability. At the beginning of the workshops, moderators presented the requirements and evidence for MRs in community pharmacies to establish a shared understanding of the intervention and its context. Experts subsequently completed an online questionnaire (LimeSurvey, Version 6.14.0, LimeSurvey GmbH, Australia) to inform the next study phase. A second questionnaire was distributed before the second workshop round in May 2025. Conceptboard® (Conceptboard Cloud Service GmbH, Stuttgart, Germany) was used to facilitate brainstorming and visualization.

### Overview of approaches taken to operationalize each outcome

**Fig. 1:**
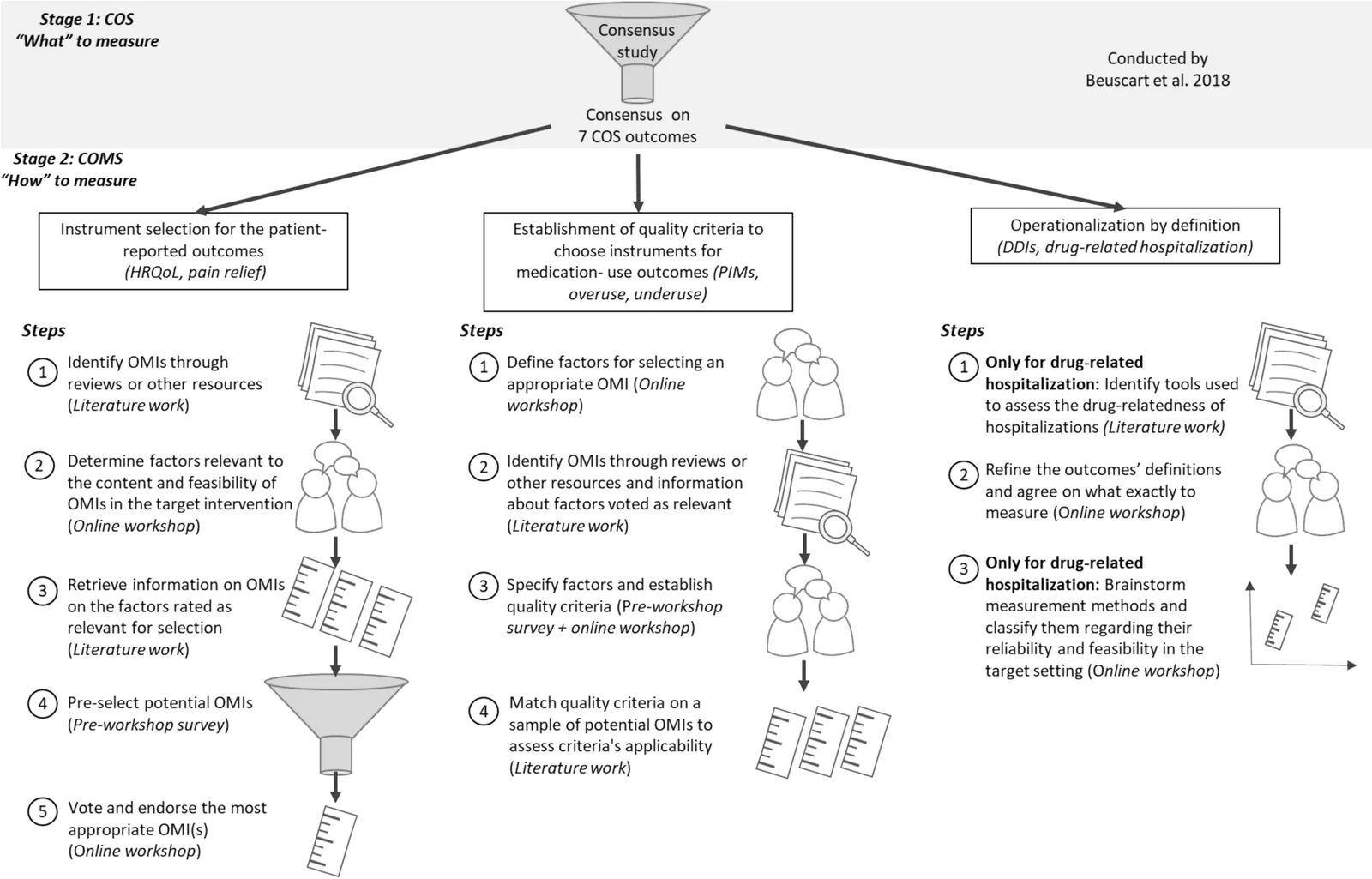
Measures undertaken to develop a Core Outcome Measurement Set for community pharmacist-led medication review studies. Different approaches were taken depending on the outcome type in the Core Outcome Set: Specific instruments were chosen for patient-reported outcomes, quality criteria for the instrument selection to measure overuse, underuse, and potentially inappropriate medications were established, and clinically significant drug-drug interactions (DDIs) and drug-related hospitalization were operationalized by refining the outcomes’ definitions of what to measure. For each category, appropriate methodological techniques were employed, including literature reviews, online surveys and workshops with an expert group. *COMS core outcome measurement set, *COS* core outcome set, *DDI* drug-drug interaction, *HRQoL* health-related quality of life, *OMI* outcome measurement instrument, *PIM* potentially inappropriate medication

Based on the outcome (type), different methodological approaches were undertaken (Fig. 1).

### Methodological approach to select the most appropriate OMIs for HRQoL and pain relief

The selection of OMIs for HRQoL and pain relief comprised five steps (Fig. 1): Step 1 involved conducting systematic searches of PubMed, Embase, and the COSMIN databases between March 2023–August 2024 to identify relevant systematic reviews of OMIs. The search strategy is presented in Supplementary file 2. To ensure that newly developed or validated OMIs were also captured, the same search strategy was subsequently applied to identify studies published after the search period covered by the most recent review. Screening was conducted independently by two reviewers, with any disagreements resolved through discussion.

In line with the COSMIN and OMERACT guidelines, Step 2 activities required a systematic evaluation of the methodological quality of potential OMIs. However, no validation studies were identified for the COS target population (older patient with multimorbidity and polypharmacy) [19]. Broadening the search to older patients in general yielded in an unmanageable number of hits, and further refinement, despite consultation with a search expert and the COSMIN team, did not sufficiently reduce the yield. Thus, an intermediate step was introduced to identify practical OMI selection factors. During the first workshop, experts discussed and rated potential factors (e.g., completion time, available languages, cost, and copyright). Anonymous voting and, where necessary, further discussion were used to achieve agreement. Factors receiving ≥ 60% agreement were retained, a threshold chosen to capture a broad yet meaningful set of selection factors and consistent with previous consensus-based research [20, 21].

In Step 3, information on the retained factors was retrieved from the literature and summarized for each OMI. Heatmaps were created to facilitate comparison of the OMIs, ranking OMIs to one another across all factors. In Step 4, the summarized information on the OMIs was distributed through the second survey, in which the experts selected the OMIs they considered most appropriate. Survey responses were analyzed descriptively and used to rank OMIs according to expert preferences. In Step 5, the three highest-ranked OMIs were presented during the second workshop. Through structured discussion, experts sought to reach agreement (100%) on the most appropriate OMI.

### Methodological approach to establish quality criteria for OMIs to measure overuse, underuse and PIMs

To operationalize overuse, underuse, and PIMs, universal quality criteria were established to guide the OMI selection. These criteria define the minimum standards an OMI must meet to be considered appropriate. This approach comprised five steps (Fig. 1): In Step 1, experts identified factors relevant to OMI selection. An initial set of factors (e.g., development method, completion time) was presented, and experts were invited to propose additional ones. Experts discussed and anonymously rated their relevance (≥ 60% agreement). The results were then reviewed in an open group discussion, and factors with ambiguous ratings were re-evaluated to clarify their relevance. In Step 2, a purpose-driven search of published literature reviews [22–25] was conducted to identify and gather information about relevant OMIs. Consistent with the COS population, only OMIs addressing older patients (≥ 65 years) were considered. Additionally, only OMIs formulated as explicit criteria—defined as clearly specified rules or measurable threshold—were included (e.g., the STOPP/START criteria [26]). Implicit criteria, such as the Medication Appropriateness Index [27], were excluded because they rely heavily on clinical judgment and individualized assessment, which limits comparability across studies. In Step 3, the OMIs were characterized according to the retained selection factors. During the second survey, the experts assessed which content should be included in the OMIs to ensure consistency with the COS definitions [19]. They also evaluated acceptable thresholds for validity and feasibility of the OMIs, such as the maximum allowable completion time. Responses were summarized descriptively. In Step 4, the experts discussed the survey results during the second workshop to reach agreement on the specification of selection factors and thereby, establish quality criteria for OMI selection. They further discussed whether separate or combined OMIs should be used. In Step 5, the established quality criteria were piloted on a sample of OMIs to assess their applicability to existing OMIs.

### Methodological approach to refine the definitions of how to measure clinically significant DDIs and drug-related hospitalization

The operationalization of clinically significant DDIs comprised a single step (Fig. 1): In the COS, DDIs were definedclinically significant when ‘having a severity rating according to the drug interaction compendia used in the study’ [19]. However, severity ratings are not standardized and may differ across different drug information resources [28]. To address this, moderators presented the standardized definition of DDI severity ratings (categorized in minor/moderate/major) proposed by Shariff et al. [28] during the second workshop. Experts then selected which severity rating should be considered clinically significant in MR studies.

To operationalize drug-related hospitalization, three steps were undertaken (Fig. 1): In Step 1, prior to the workshop, a search was conducted to identify well-established or validated tools for assessing whether a hospitalization was drug-related. In Step 2, experts discussed and refined the outcome definition. In Step 3, experts identified potential methods to collect hospitalizations in general and classified them according to their reliability and feasibility in community pharmacist-led MR studies.

### Determination of frequency and timing for outcome measurement

As part of the first survey, the experts were asked to specify the minimum frequency and timing of outcome measurement. They also proposed appropriate measurement time points relative to study’s baseline. The survey results were descriptively summarized, discussed during the second workshop, and used to reach agreement.

### Ethics approval

The study was approved by the Ethics Committee of the Medical Faculty of Heidelberg University (S-444/2023) on 16 August 2023.

## Results

### Experts

The expert group was formed of ten participants; one participant dropped out after the first workshop. Six experts attended the second workshop. Due to the second survey—which was completed by eight experts—the views of those who could not attend could also be included. They had diverse professional backgrounds and represented six European countries. Some participants indicated that they had already conducted MRs themselves (*n* = 4/10, 2 answers missing) or participated in the evaluation of MR study (*n* = 4/10, 2 answers missing; in Supplementary file 3).

### Selection of the most appropriate OMI for the patient-reported outcomes

The review process as displayed in the PRISMA flow diagram in Supplementary file 4 retrieved a total of 17 HRQoL instruments from the literature. Selection factors were: content (*n* = 10/10 votes), length (*n* = 7/10), completion time (*n* = 8/10), availability of local languages (*n* = 7/10), validation in the targeted patient population (*n* = 10/10), copyright (*n* = 8/10) and, if applicable, additional equipment required (*n* = 6/10). The experts agreed that it should generally be determined whether validation was available. Accordingly, it was checked whether the OMI was validated in general, for older people or those on polypharmacy. The created heatmaps as well as further information on the OMIs are shown in Supplementary file 5. This selection process resulted in the choice of the EuroQoL Five Dimensions 5 Levels (EQ-5D-5L) [29] due to its widespread use, availability in many languages, ease of use, and it is freely available upon registration. The experts recognized few limitations of the EQ-5D-5L, as the recall period of one day might be very brief and the questions had a more generic focus.

To measure pain relief, nine potential OMIs were identified (PRISMA flow diagram in Supplementary file 4 and heatmap in Supplementary file 5). Further information on the OMIs is shown in Supplementary file 5. The discussion among the experts concluded that both the visual analogue scale (VAS) and the numerical rating scale (NRS) were equally appropriate for use in MR studies, as they were simple, commonly used and license-free. Regardless of the scale used, it was recommended that pain ratings should be converted to a standardized numerical score from 0 to 10, where 0 indicated no pain and 10 the worst imaginable pain.

### Established quality criteria to select OMIs to measure overuse, underuse and PIMs

In total, 23 OMIs were retrieved that measured overuse, underuse, and/or PIMs. Relevant selection factors for an appropriate OMI were: content (*n* = 10/10 votes), method(s) used to develop or validate the OMI (*n* = 6/10), completion time (*n* = 9/10), required level of expertise to complete OMI (*n* = 7/10), up-to-datedness (*n* = 6/10), and copyright status (*n* = 8/10). In Supplementary file 5 information on the selection factors for the identified OMIs is presented.

Table 1 summarizes the established quality criteria for OMI selection. Experts agreed that the content-related criteria should meet the outcomes’ definition as defined by the COS [19]. Overuse is defined as ‘the use or prescription of more drugs than are clinically necessary, including: (1) any drug prescribed or used without an evidence-based clinical indication; (2) therapeutic duplication; and (3) medication prescribed or used beyond the recommended duration.’[19] This means that an appropriate OMI for overuse should contain ‘stopping inappropriate medication’, ‘therapeutic duplication’ and ‘duration of therapy’. The results of the pilot testing of selected content-related quality criteria on a sample of OMIs are shown in Supplementary file 6. The OMIs matched the criteria to a varying degree. In addition, validity- and feasibility-related criteria were defined. However, no agreement was reached on standards for these criteria, and fixed thresholds for quantifiable criteria (e.g., maximum acceptable application time) could not be established.

**Table 1:** Quality criteria to measure overuse, underuse, and potentially inappropriate medications (PIMs)

*Quality criteria related to the content*	
	*Specification of quality criteria: linked core outcomes and additional considerations*
Stopping inappropriate medication	• Important for overuse
Starting appropriate medication	• Important for underuse
Alternatives suggested	• Important for PIMs
Dosing	• Including the impact of organ impairment (e.g., of the liver or kidneys) on dosing
Duration of therapy	• Important for overuse and underuse
Therapeutic duplications	• Important for overuse
Drug-disease interactions	• Important for PIMs, not as important for overuse/underuse
*Quality criteria related to the validity and feasibility*	
	*Specification of quality criteria*
Up-To-Datedness	• OMI should be as up-to-date as possible
Development/validation method	• The development/validation of the OMI should be clearly described
	• E.g., Delphi consensus, expert consensus and prospective study are appropriate methods
Completion time	• The OMI with the shortest completion time should be chosen, if all other OMIs are identical in every other aspect
Terms of use	• If possible, OMI should be free to use
Who should be able to fill it out	• All healthcare professionals included in the study should be able to complete the OMI
	• Healthcare professionals and researchers who are following a manual/training should be able to complete the OMI
Ease of use	• OMI should be easily to use in daily practice
Digital format	• If possible, a digital format of the OMIs is preferred

### Standardized definition to measure clinically significant DDIs and drug-related hospitalization

Experts decided to limit the operationalization of the outcome on DDI to ‘major’ DDIs, as their measurement was more specific. A major DDI is defined as an interaction that may result in a life-threatening situation and/or cause a significant hazard and/or carry a high risk of dangerous outcome, for which concurrent use is contraindicated [28]. The experts suggested that future studies should also report the interventions implemented to prevent the effects of DDIs, or, if no action was taken, provide the reasons. Finally, participants preferred the term ‘potential clinically significant DDIs’ because the clinical effect of a DDI may vary between patients.

No well-established tools for assessing the drug-relatedness of hospitalizations were identified. Consequently, no specific methodological requirements were defined for determining drug-relatedness. To fulfil the COS, this assessment would have to be conducted at the discretion of the study authors. As an interim-solution, the experts agreed to include all-cause hospitalizations as an additional outcome, as these can be measured in a standardized way.

**Fig. 2:**
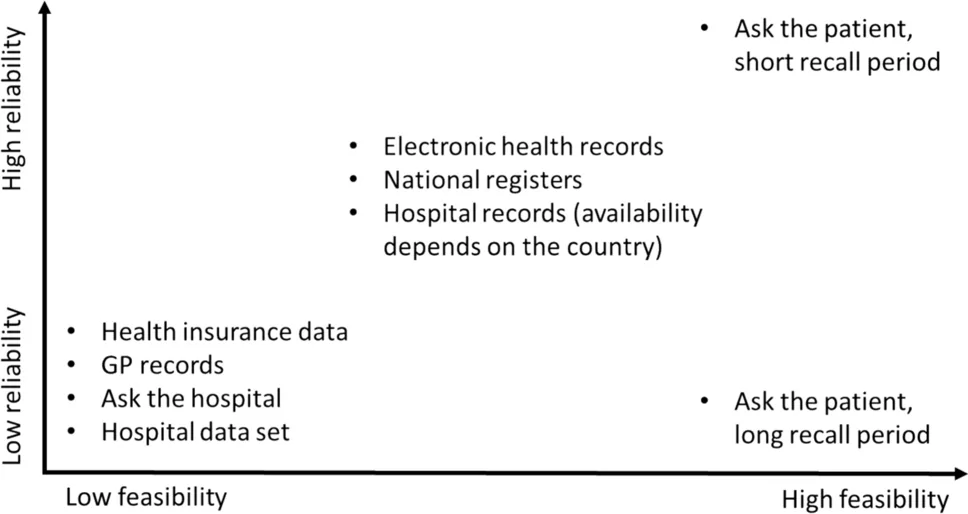
Methods to measure drug-related and all-cause hospitalizations in community pharmacy studies classified by their reliability and feasibility. GP = General practitioner

The experts further refined the definition of hospitalization in general as at least one overnight stay (excluding, e.g., visits to the emergency department that do not lead to an inpatient stay). They also refined the outcome to only unplanned admissions. This definition can be applied to both drug-related and all-cause hospitalizations. In the community pharmacy setting, the experts agreed that patient self-report, when collected within an appropriate recall period, was the only measurement method that combined high feasibility with high reliability (Fig. 2). It was therefore considered the most appropriate method for measuring hospitalization in this context and is applicable to both drug-related and all-cause hospitalizations. They also agreed that a six-month recall period was acceptable, as a hospitalization was considered significant and, therefore, memorable event.

### Standardized frequency and timing for outcome measurement

**Fig. 3:**
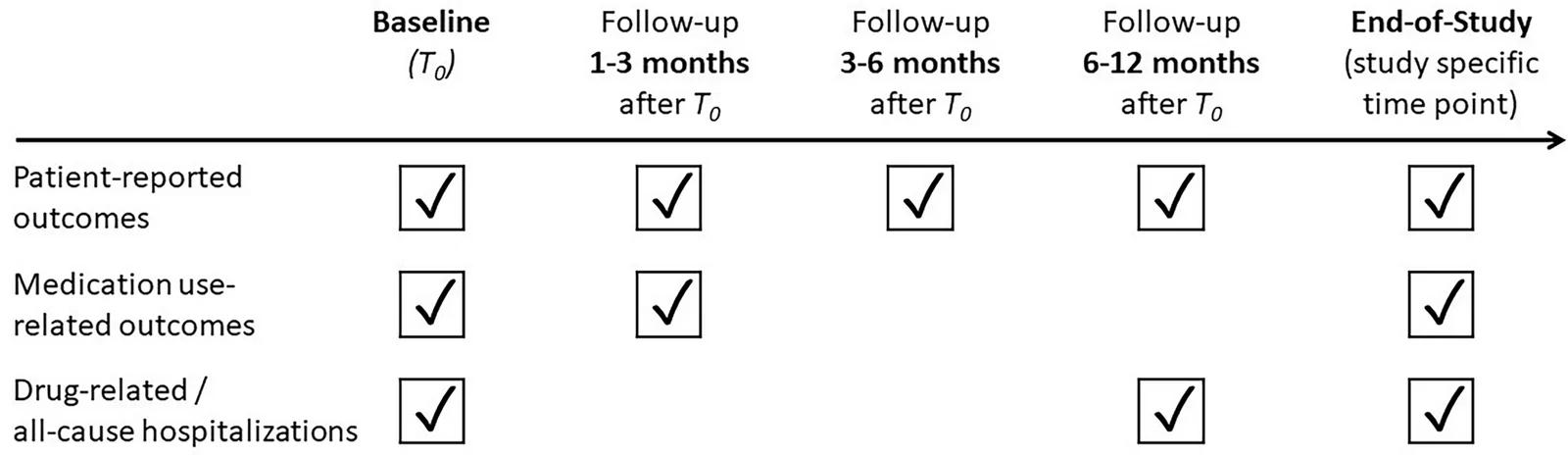
Standardized frequency and timing for outcome measurement in medication review studies, categorized by outcome type

Experts agreed on a minimum of five standardized time points for patient-reported outcomes, on at least three for outcomes related to medication use and three for hospitalization. They also defined time points/ranges in relation to study baseline for when these measurements should be conducted (Fig. 3).

### Final COMS for MR studies in community pharmacies

The final COMS is presented in Table 2.

**Table 2:** Final Core Outcome Measurement Set (COMS) for medication review studies conducted in community pharmacies

Core outcome	Proposed way for measurement	Proposed time points/ranges for measurement
*Patient-reported outcomes*		
Health-related quality of life (HRQoL)	Using the EQ-5D-5L	Baseline
		Follow-up after 1–3 months
		Follow-up after 3–6 months
		Follow-up after 6–12 months
		End-of-study
Pain relief	Using visual or numerical rating scale (results conversed to a 0–10-score)	Baseline
		Follow-up after 1–3 months
		Follow-up after 3–6 months
		Follow-up after 6–12 months
		End-of-study
*Medication use*		
Overuse	In terms of content, OMI should include: stopping inappropriate medication, duration of therapy, therapeutic duplication	Baseline
		Follow-up after 1–3 months
		End-of-study
Underuse	In terms of content, OMI should include: starting medications, duration of therapy	Baseline
		Follow-up after 1–3 months
		End-of-study
Potentially inappropriate medications (PIMs)	In terms of content, OMI should include: alternatives suggested, drug-disease interactions	Baseline
		Follow-up after 1–3 months
		End-of-study
Potential clinically significant drug-drug-interactions (DDIs)	Only DDIs with ‘major’ severity rating should be measured	Baseline
		Follow-up after 1–3 months
		End-of-study
*Adverse event*		
Drug-related/all-cause hospitalization	At least one overnight stay and should be measured by patient report every six months For drug-related admissions, the causality assessment method/tool used should be specified	Baseline
		Follow-up after 6–12 months
		End-of-study

*OMI* Outcome measurement instrument

## Discussion

This study operationalized a previously published COS for MR studies [19] in the community pharmacy setting by identifying the most appropriate OMIs for HRQoL and pain relief, establishing quality criteria to guide OMI selection for overuse, underuse and PIMs, and refining outcome definitions for clinically significant DDIs and drug-related hospitalization. By combing systematic literature searches of OMIs with expert involvement, the final COMS is both evidence-informed and aligned with current research. Thus, this COMS may help mitigate heterogeneity in MR studies, which currently impedes evidence synthesis [6, 30–32].

The COSMIN/OMERACT initiatives recommend selecting one OMI for each core outcome [18]. This was achieved for the two patient-reported outcomes: The EQ-5D-5L is a very brief OMI to measure HRQoL and covers five domains: mobility, self-care, usual activities, pain/discomfort, and anxiety/depression [29]. It is widely used due to available translations into more than 150 languages and its short completion time. The EQ-5D-5L has demonstrated validity, responsiveness and feasibility across diverse populations and diseases [33] and its use is well-established in older patients with substantial multimorbidity and polypharmacy [34], a population likely to benefit from a MR. Nevertheless, its narrower focus could lead to some aspects of HRQoL being missed, e.g., it failed to address how side effects affected HRQoL [35]. Although OMIs for medication-related quality of life are now available, they largely target populations with specific health conditions [36]. Since the COS definition explicitly refers to HRQoL in general, disease-specific or medication-specific OMIs were not pursued. Nevertheless, it may be necessary to consider supplementing the EQ-5D-5L with an additional medication-related question.

For pain relief, both the NRS and VAS were considered appropriate and can be used interchangeably after conversion to a 0–10 NRS score. Both OMIs demonstrate comparable performance [37], however, older adults, including those with mild to moderate cognitive impairment, prefer the NRS [38]. The NRS offers practical advantages as it can be administered verbally [38, 39]. Typically, the time frame for VAS and NRS assessments refers to pain experienced over the past 24 h [39]; this reference period should remain consistent. However, both the NRS and VAS have limitations—their scores can be influenced by various cognitive and contextual factors [40]. These OMIs should only be used to assess changes in individuals over time rather than for direct comparisons between patients. Also, pain may not be a relevant outcome for all patients included in MR studies. Therefore, it should first be assessed whether pain is a relevant presenting symptom.

Experts agreed on the content-related quality criteria for the appropriateness of using OMIs to measure medication-use outcomes. However, criteria concerning the validity or feasibility of an OMI, such as completion time or up-to-datedness, could not be comprehensively specified because they are interdependent and context-dependent. These criteria can therefore only be considered when multiple content-valid instruments are available. The applicability of OMIs is also likely to vary across healthcare systems, meaning some variability will remain. The assessment of PIMs represents a subset of drug-related problems that can indicate overuse of inappropriate medications. To reduce the burden on researchers and pharmacists, preference should be given to OMIs covering multiple core outcomes.

The available drug information resources differ in terms of the DDIs they include and severity assessment [28, 41, 42], therefore, variability in DDI measurement is unavoidable. Limiting measurement to major DDIs—those that might result in a clinically significant change in effectiveness or safety and require therapy modifications—was deemed most specific and practical. In the original COS, no distinction was made between actual and potential DDIs. The term ‘potential’ was included, as not all DDIs manifest clinically. It was also recommended to document any measures taken to address the DDI, allowing for assessment of actions taken or omitted. When measuring DDI, it is also important to consider the quality of the underlying resources. Although this study did not specify such criteria; examples that may support resource selection are a DDI management checklist [43] and the list of 66 potentially clinically significant DDIs in older patients [44]. For pharmacy practice, an assessment tool to evaluate the quality of DDI software resources is available [45].

The COS [19] originally defined hospitalization as drug-related hospital admissions. However, during the course of this study, it became evident that while tools to estimate drug-relatedness such as the AT-HARM10 tool [46] exist, they lack widespread implementation. As a result, drug-relatedness cannot currently be measured consistently across studies. The selection of an appropriate tool will therefore remain at the discretion of the study authors and should be reported transparently. To address this limitation, the experts recommended including all-cause hospitalization as an additional outcome. Furthermore, a general definition of hospitalization was established that can be applied to both all-cause and drug-related hospitalizations. Hospitalizations are defined as unplanned admissions involving at least one overnight stay, in line with official references [47].

The recall period was chosen for practical reasons as a compromise between feasibility and reliability and set at six months. While it is well-known that longer recall periods reduce accuracy [48, 49], major events are unlikely to be entirely forgotten [50]. However, respondents tend to recall events as having occurred more recently than they actually did, which can lead to overestimation. Temporal anchors have been suggested, such as the last visit.

### Future developments

The final COMS reflects a pragmatic balance between evidence and feasibility, which is essential for widespread adoption across studies. However, this COMS may require future updates as more appropriate OMIs become available, particularly for outcomes for which appropriate OMIs are currently lacking, such as drug-related hospitalizations. Future work should focus on the feasibility of COMS implementation in community pharmacy studies. Due to the variability in MR practices, it will also be necessary to define the core components of a MR intervention.

Additionally, the limited uptake of the MR COS is likely influenced by multiple factors. While the outcome measurement guidance provided by the developed COMS may help address one important barrier, it is unlikely to fully resolve the low adoption of the MR COS. Future efforts should therefore also address other barriers, including limited awareness of the MR COS. Likewise, the study team will actively promote COMS uptake through dissemination, stakeholder engagement, collaboration with professional societies and conference presentations, consistent with recommendations for COS implementation in general [15, 51].

### Limitations

This study has a number of limitations. Firstly, the COSMIN/OMERACT recommendations had to be modified for the systematic evaluation of the methodological quality of each OMI for the patient-reported outcomes. The present approach (i.e., emphasizing also other practical selection factors) was chosen because strict search filters (older patients with polypharmacy who had undergone a MR) yielded no results for validation studies. However, the general validity OMIs was checked in older patients and in patients with polypharmacy. Secondly, according to the guidelines, a consensus process should be employed to reach a decision on OMIs. For this work, a small but diverse group of experts was assembled that enabled productive and goal-oriented discussion. Although no substantially new OMIs or quality criteria emerged during the later stages of the process, and the participating experts continued to represent the predefined areas of expertise, additional experts may have contributed alternative perspectives. While efforts were made to ensure broad representation, only experts from European countries were included. This may have introduced cultural bias and could limit generalizability beyond Europe. However, throughout the development process, particular emphasis was placed on selecting OMIs with broad international applicability. Nevertheless, further validation of the COMS involving a more geographically diverse international expert panel would strengthen its relevance. Thirdly, no suitable tool for assessing drug-relatedness in hospitalizations could be identified. Consequently, the selection of assessment methods will be left to the individual study authors, which is likely to introduce heterogeneity in outcome measurement. The inclusion of all-cause hospitalizations as an additional outcome is considered a pragmatic interim solution rather than the final preferred measurement approach. While all-cause hospitalization can be measured in a standardized manner, it lacks specificity and may be influenced by factors unrelated to MRs. Future efforts should prioritize the development, validation, and implementation of practical methods for assessing drug-related hospitalizations, thereby allowing the COMS to be updated in line with the original intent of the COS. Lastly, the absence of patient involvement in the development of this COMS represents an important limitation. Patient representatives could have provided valuable insights into the acceptability, relevance, and feasibility of the recommended patient-reported OMIs. Although the selection of OMIs was guided by the aim of minimizing patient burden, direct patient input was not obtained. Future work should therefore incorporate patient representatives to evaluate the acceptability of the COMS and inform its ongoing refinement.

## Conclusion

This COMS operationalized the pre-existing COS and provides a standardized framework based on expert agreement to guide outcome measurement in community pharmacist-led MR studies. Its adoption may enhance comparability across studies, improve quality of evidence synthesis, and inform better decision-making in both clinical and policy domains. Encouraging adoption through professional networks, integration into relevant databases, and inclusion in funding and journal requirements will be critical implementing this COMS.

## Supplementary Information

Supplementary file 1: Questionnaires.

Supplementary file 2: Search strategy.

Supplementary file 3: Sociodemographic Data.

- Sociodemographic data of the expert panel (N = 8*).

Supplementary file 4: PRISMA flow diagrams, including:

- Prisma flow diagram for the search of health-related quality of life instruments.
- Prisma flow diagram for the search of pain relief instruments.

Supplementary file 5: Heatmaps and overviews of HRQoL, pain relief, underuse and PIM instruments, including:

- Heatmap of health-related quality of life instruments.
- Overview of health-related quality of life instruments.
- Heatmap of pain relief instruments.
- Overview of pain relief instruments.
- Overview of instruments related to overuse, underuse and potentially inappropriate medications (PIMs).

Supplementary file 6: Content-related quality criteria tested on a sample of instruments for overuse, underuse and PIMs. *Overview of instruments and extent to which they match the established quality criteria.* Including:

- Recommended content-related quality criteria of appropriate outcome measurement instruments (OMIs) for overuse, underuse and/or PIMs piloted on a sample of OMIs.

Below is the link to the electronic supplementary material.

Supplementary file1 (PDF 341 KB)

Supplementary file2 (PDF 252 KB)

Supplementary file3 (PDF 257 KB)

Supplementary file4 (PDF 407 KB)

Supplementary file5 (XLSX 16177 KB)

Supplementary file6 (PDF 370 KB)

## Data Availability

All data generated or analyzed during this study are included in this published article and its supplementary information files.
