# Supplementary material for: Development of a Core Outcome Measurement Set for studies of community pharmacist-led medication reviews in older patients with multimorbidity and polypharmacy
Source: Int J Clin Pharm. 2026 Sep 16;48(6):2467–78. doi: 10.1007/s11096-026-02223-y (PMC13731522; doi:10.1007/s11096-026-02223-y)
Supplement: Supplementary file 1 [file 11096_2026_2223_MOESM1_ESM.pdf]

Development of a Core Outcome Measurement Set for studies of community pharmacy-led medication reviews in older patients with multimorbidity and polypharmacy

Cathrin J. Vogt[1], Christiane Eickhoff[2], Veerle Foulon[3], Carmel Hughes[4], Jens Kessler[5], Cinara Paul[6,7], Cristín Ryan[8], Martin Schulz[2,9], Martina Teichert[10], Ronja Woltersdorf[11], Viktoria S. Wurmbach[1], Hanna M. Seidling[1]*

[1]Heidelberg University, Medical Faculty Heidelberg / Heidelberg University Hospital, Medical Clinic, Internal Medicine IX – Department of Clinical Pharmacology and Pharmacoepidemiology, Cooperation Unit Clinical Pharmacy, Im Neuenheimer Feld 410, 69120, Heidelberg, Germany.

[2]Department of Medicine, ABDA-Federal Union of German Associations of Pharmacists, Berlin, Germany.

[3]Department of Pharmaceutical and Pharmacological Sciences, KU Leuven, Belgium.

[4]School of Pharmacy, Queen's University Belfast, Belfast, Northern Ireland.

[5]Medical Faculty Heidelberg, Department of Anaesthesiology, Pain Medicine Section, Heidelberg University, Heidelberg, Germany.

[6]Department of General Internal Medicine and Psychosomatics, University Hospital Heidelberg, Heidelberg, Germany.

[7]Institute for General Practice and Interprofessional Care, Tübingen University Hospital, Tübingen, Germany.

[8]School of Pharmacy and Pharmaceutical Sciences, Trinity College Dublin, Republic of Ireland.

[9]Institute of Pharmacy, Freie Universität Berlin, Berlin, Germany.

[10]Primary Health Care, Leiden University Medical Center, Leiden, The Netherlands.

[11]Department of Clinical Pharmacy, Institute of Pharmacy, University of Bonn, Bonn, Germany.

*Corresponding author:

Heidelberg University, Medical Faculty Heidelberg / Heidelberg University Hospital, Medical Clinic, Internal Medicine IX – Department of Clinical Pharmacology and Pharmacoepidemiology, Cooperation Unit Clinical Pharmacy, Im Neuenheimer Feld 410, 69120, Heidelberg, Germany

Mail: hanna.seidling@med.uni-heidelberg.de

Supplementary file 1. Questionnaires.

Questionnaire 1: Sociodemographic data and pre-selection of instruments

Socio-demographic and general information

Gender:

☐ Female
☐ Male
☐ Non-binary
☐ Do not want to specify

Age in years:

☐ ≤ 30 ☐ 31 – 40 ☐ 41 – 50
☐ 51 – 60 ☐ ≥ 61

In which country are you currently working?

____

What is your current profession? You can select several answers.

☐ Pharmacist ☐ Researcher
☐ Physician ☐ Other (please specify): ____________________________

To what extent do you consider your work to be practical or research-oriented?

☐ Entirely practical ☐ Mostly practical ☐ Balanced ☐ Mostly Research ☐ Entirely Research

Are you familiar with the intervention "medication review"?

☐ No
☐ Yes → if yes: Have you already conducted a medication review?
☐ Yes ☐ No

→ if yes: Have you already evaluated a medication review?
☐ Yes ☐ No

In which of the core outcomes of medication review studies do you have expertise in? You can select several outcomes.

☐ Health-related quality of life ☐ Overuse
☐ Pain relief ☐ Underuse
☐ Potentially Inappropriate Medications (PIMs) ☐ Drug-Drug-Interactions
☐ Drug-related hospital admissions

Pre-selection of instruments

Please select your top instruments for measuring Health-Related Quality of Life in medication review studies in community pharmacies. You can select max. 5 instruments.

☐ 15D (15D instrument of health-related quality of life)
☐ AQoL-4D (Assessment of Quality of Life (AQoL)-4D Basic)
☐ AQoL-6D (Assessment of Quality of Life (AQoL)-6D)
☐ AQoL-7D (Assessment of Quality of Life (AQoL)-7D)
☐ AQoL-8D (Assessment of Quality of Life (AQoL)-8D)
☐ DHP (Duke Health Profile)
☐ EQ-5D-3L (EuroQoL Five Dimensions 3L)
☐ EQ-5D-5L (EuroQoL Five Dimensions 5L)
☐ HUI-2 (Health Utilities Index-2)
☐ HUI-3 (Health Utilities Index-3)
☐ NHP (Nottingham Health Profile)
☐ SF-12 (12-Item Short Form Health Survey)
☐ SF-36 (36-Item Short Form Health Survey)
☐ SF-6D (6-Dimension Short Form Health Survey, version 2)
☐ SF-8 (8-Item Short Form Health Survey)
☐ Stark QoL
☐ TAAQOL (TNO-AZL Questionnaire for Adult's Health-Related Quality of Life)

Please select your top instruments for measuring pain relief in medication review studies in community pharmacies. You can select max. 3 instruments.

☐ BPI (Brief Pain Inventory)
☐ BPI - Short Form (Brief Pain Inventory)
☐ CPGS (Chronic Pain Grade Scale)
☐ McGill Pain Questionnaire - Long Form
☐ McGill Pain Questionnaire - Short Form
☐ VAS (Visual Analogue Scale)
☐ VRS (Verbal Rating Scale)
☐ PROMIS Pain Intensity - Short Form

Questionnaire 2: Pre-selection of instruments and of frequency / timing for outcome measurement

Patient-reported outcomes

Based on our latest shortlist of instruments for measuring ***Health-Related Quality of Life*** and **pain relief**, we selected the top contenders. To narrow down the options further, we kindly ask you to choose your favorite instrument from the list and briefly explain why you chose it.

Please choose your preferred instrument for measuring *Health-Related Quality of Life* (The vote results are provided in parentheses). Choose one of the following answers.

☐ EQ-ED-5L (n=6/8)
☐ EQ-ED-3L (n=4/8)
☐ SF-36 (n=6/8)
☐ SF-12 (n=4/8)
☐ SF-8 (n=3/8)
☐ No preference

Please briefly justify your answer:

____

Please choose your preferred instrument for measuring *pain relief* (The vote results are provided in parentheses). Choose one of the following answers.

☐ Visual Analogue Scale (VAS) (n=7/8)
☐ Numerical Rating Scale (NRS) (n=4/8)
☐ Brief Pain Inventory (Short Form) (n=3/8)
☐ No preference

Please briefly justify your answer:

____

Overuse, Underuse and Potentially Inappropriate Medications (PIMs)

For the outcomes of ***overuse, underuse, and Potentially Inappropriate Medications (PIMs),*** we aim to define quality criteria that enable researchers to assess whether the instruments are appropriate for their intended use. This approach also takes into account study or country specific prerequisites.

Some instruments may measure multiple outcomes, i.e. overuse, underuse and PIMs, at the same time. Would you prefer to use an instrument that combines multiple outcomes if the other quality criteria are also met?

☐ Yes
☐ No
☐ No preference

In your opinion, how important is it that these <u>content-related quality criteria</u> are considered in each instrument to measure overuse, underuse and/or PIMs?

	Not important	Important	Very important	No preference
Stopping inappropriate medications				
Starting appropriate medications				
Alternatives suggested				
Considers organ impairments				
Dosing				
Duration of therapy				
Drug-disease interactions				

What it the <u>maximum completion time</u> that is still appropriate for each instrument?

☐ < 15 minutes
☐ 15-30 minutes
☐ 30-45 minutes
☐ > 60 minutes
☐ Other: _________
☐ No preference

If you think there is a difference between the outcomes overuse, underuse and PIMs in this quality criterion, please specify here:

____

What is an appropriate validation method for the development of each instrument?

☐ Delphi consensus / RAND-UCLA consensus

☐ Expert consensus (i.e., expert group discussion using, e.g., nominal group technique)

☐ Prospective study

☐ Other: _________

☐ No preference

If you think there is a difference between the outcomes overuse, underuse and PIMs in this quality criterion, please specify here:

____

What is the longest period of time that would be appropriate for the last update of each instrument?

☐ < 5 years

☐ 5-10 years

☐ > 10 years

☐ Other: _________

☐ No preference

If you think there is a difference between the outcomes overuse, underuse and PIMs in this quality criterion, please specify here:

____

What are appropriate terms of use for each instrument?

☐ Not restricted by copyright

☐ Licensed, use requires permission from the developer, but is free for non-commercial use

☐ Licensed and user fee

☐ Other: _________

☐ No preference

If you think there is a difference between the outcomes overuse, underuse and PIMs in this quality criterion, please specify here:

____

Who should be able to perform the assessment with each instrument?

☐ Researchers

☐ Pharmacists included in the study

☐ Other: _________

☐ No preference

If you think there is a difference between the outcomes overuse, underuse and PIMs in this quality criterion, please specify here:

____

Should there be a digital format (e.g., tablet-based) of each instrument?

☐ Yes

☐ No

☐ No preference

If you think there is a difference between the outcomes overuse, underuse and PIMs in this quality criterion, please specify here:

____

Drug-Drug interactions

Drug-drug interactions (DDIs) are likely to be identified and evaluated using interaction databases. Because there are different definitions of DDIs, it is important to clarify your understanding of which DDIs should be considered 'clinically important'. This will help researchers to verify that the resource is appropriate for measuring DDIs in medication review studies ion community pharmacies.

From a pharmacodynamic point of view, for which adverse events should drug-drug interactions be la as 'clinically significant'?

☐ Exacerbate the patient's condition or lead to a change in therapy

☐ Require monitoring or adjustment of dosages but are not typically life-threatening

☐ Result in severe adverse effects, including toxicity, organ failure, or fatal outcomes

☐ Necessitate avoiding the drug combination or implementing strict monitoring

☐ Do not usually require adjustment of therapy, but lead to a change in AUC

☐ Other: _________

☐ No preference

Please briefly justify your answer::

____

Hospitalization

Based on the results of our first workshop, the only method that was considered reliable and feasible for measuring hospitalization in our intended intervention was to interview the patient within a short period of time.

What would be an appropriate recall period for patients to confirm whether they have been hospitalized? Choose one of the following answers.

☐ Within the last 4 weeks
☐ Within the last 6 weeks
☐ Within the last 8 weeks
☐ Within the last 10 weeks
☐ Other: _________
☐ No preference

Time points for measurement

Since different time points for measurement might lead to incomparable study results, we also want to define the appropriate time points that should be taken as a minimum. You can choose how many time points you consider appropriate, starting with study initiation.

How often should the <u>patient-reported outcomes</u> (Health-Related Quality of Life and pain relief) at least be measured? Please also specify when the measurement should take place relative to the study's start.

☐ Baseline (T0)
☐ Follow-up 1: (Please specify how many months after T0): _________
☐ Follow-up 2: (Please specify how many months after T0): _________
☐ Follow-up 3: (Please specify how many months after T0): _________
☐ Follow-up 4: (Please specify how many months after T0): _________
☐ End-of study visit: (Please specify how many months after T0):_________
☐ Other: _________
☐ No preference

How often should the medication use-related outcomes (overuse, underuse, PIMs, DDIs) at least be measured? Please also specify when the measurement should take place relative to the study's start.

☐ Baseline (T0)
☐ Follow-up 1: (Please specify how many months after T0): _________
☐ Follow-up 2: (Please specify how many months after T0): _________
☐ Follow-up 3: (Please specify how many months after T0): _________
☐ Follow-up 4: (Please specify how many months after T0): _________
☐ End-of study visit: (Please specify how many months after T0):________
☐ Other: _________
☐ No preference

How often should hospitalization at least be measured? Please also specify when the measurement should take place relative to the study's start.

☐ Baseline (T0)
☐ Follow-up 1: (Please specify how many months after T0): _________
☐ Follow-up 2: (Please specify how many months after T0): _________
☐ Follow-up 3: (Please specify how many months after T0): _________
☐ Follow-up 4: (Please specify how many months after T0): _________
☐ End-of study visit: (Please specify how many months after T0):________
☐ Other: _________
☐ No preference
