# Supplementary material for: Development of a Core Outcome Measurement Set for studies of community pharmacist-led medication reviews in older patients with multimorbidity and polypharmacy
Source: Int J Clin Pharm. 2026 Sep 16;48(6):2467–78. doi: 10.1007/s11096-026-02223-y (PMC13731522; doi:10.1007/s11096-026-02223-y)
Supplement: Supplementary file 2 [file 11096_2026_2223_MOESM2_ESM.pdf]

Development of a Core Outcome Measurement Set for studies of community pharmacy-led medication reviews in older patients with multimorbidity and polypharmacy

Cathrin J. Vogt[1], Christiane Eickhoff[2], Veerle Foulon[3], Carmel Hughes[4], Jens Kessler[5], Cinara Paul[6,7], Cristín Ryan[8], Martin Schulz[2,9], Martina Teichert[10], Ronja Woltersdorf[11], Viktoria S. Wurmbach[1], Hanna M. Seidling[1]*

[1]Heidelberg University, Medical Faculty Heidelberg / Heidelberg University Hospital, Medical Clinic, Internal Medicine IX – Department of Clinical Pharmacology and Pharmacoepidemiology, Cooperation Unit Clinical Pharmacy, Im Neuenheimer Feld 410, 69120, Heidelberg, Germany.

[2]Department of Medicine, ABDA-Federal Union of German Associations of Pharmacists, Berlin, Germany.

[3]Department of Pharmaceutical and Pharmacological Sciences, KU Leuven, Belgium.

[4]School of Pharmacy, Queen's University Belfast, Belfast, Northern Ireland.

[5]Medical Faculty Heidelberg, Department of Anaesthesiology, Pain Medicine Section, Heidelberg University, Heidelberg, Germany.

[6]Department of General Internal Medicine and Psychosomatics, University Hospital Heidelberg, Heidelberg, Germany.

[7]Institute for General Practice and Interprofessional Care, Tübingen University Hospital, Tübingen, Germany.

[8]School of Pharmacy and Pharmaceutical Sciences, Trinity College Dublin, Republic of Ireland.

[9]Institute of Pharmacy, Freie Universität Berlin, Berlin, Germany.

[10]Primary Health Care, Leiden University Medical Center, Leiden, The Netherlands.

[11]Department of Clinical Pharmacy, Institute of Pharmacy, University of Bonn, Bonn, Germany.

*Corresponding author:

Heidelberg University, Medical Faculty Heidelberg / Heidelberg University Hospital, Medical Clinic, Internal Medicine IX – Department of Clinical Pharmacology and Pharmacoepidemiology, Cooperation Unit Clinical Pharmacy, Im Neuenheimer Feld 410, 69120, Heidelberg, Germany

Mail: hanna.seidling@med.uni-heidelberg.de

Supplementary file 2. Search strategies for outcome measurement instruments for Health-related Quality of Life and pain relief

Exemplarily for PubMed:

For health-related quality of life: "Quality of Life"[MeSH Terms] OR "life quality"[ti] OR "Quality of Life"[ti] OR "health related quality of life"[ti] OR "health-related quality of life"[ti] OR "HRQOL"[ti]

For pain relief: Pain[ti]

AND

(((systematic review[ti] OR systematic literature review[ti] OR systematic scoping review[ti] OR systematic narrative review[ti] OR systematic qualitative review[ti] OR systematic evidence review[ti] OR systematic quantitative review[ti] OR systematic meta-review[ti] OR systematic critical review[ti] OR systematic mixed studies review[ti] OR systematic mapping review[ti] OR systematic cochrane review[ti] OR systematic search and review[ti] OR systematic integrative review[ti]) NOT comment[pt] NOT (protocol[ti] OR protocols[ti])) NOT MEDLINE [subset]) OR (Cochrane Database Syst Rev[ta] AND review[pt]) OR systematic review[pt]

AND

"instrument*"[ti] OR "scale*"[ti] OR "measure*"[ti] OR "questionnaire*"[ti] OR "tool*"[ti] OR ("outcome"[ti] AND "measurement*"[ti]) OR ("test*"[ti] AND "review"[ti])

These search strategies were subsequently adapted for use in Embase.
