# Supplementary material for: Development of a Core Outcome Measurement Set for studies of community pharmacist-led medication reviews in older patients with multimorbidity and polypharmacy
Source: Int J Clin Pharm. 2026 Sep 16;48(6):2467–78. doi: 10.1007/s11096-026-02223-y (PMC13731522; doi:10.1007/s11096-026-02223-y)
Supplement: Supplementary file 3 [file 11096_2026_2223_MOESM3_ESM.pdf]

Development of a Core Outcome Measurement Set for studies of community pharmacy-led medication reviews in older patients with multimorbidity and polypharmacy

Cathrin J. Vogt[1], Christiane Eickhoff[2], Veerle Foulon[3], Carmel Hughes[4], Jens Kessler[5], Cinara Paul[6,7], Cristín Ryan[8], Martin Schulz[2,9], Martina Teichert[10], Ronja Woltersdorf[11], Viktoria S. Wurmbach[1], Hanna M. Seidling[1]*

[1]Heidelberg University, Medical Faculty Heidelberg / Heidelberg University Hospital, Medical Clinic, Internal Medicine IX – Department of Clinical Pharmacology and Pharmacoepidemiology, Cooperation Unit Clinical Pharmacy, Im Neuenheimer Feld 410, 69120, Heidelberg, Germany.

[2]Department of Medicine, ABDA-Federal Union of German Associations of Pharmacists, Berlin, Germany.

[3]Department of Pharmaceutical and Pharmacological Sciences, KU Leuven, Belgium.

[4]School of Pharmacy, Queen's University Belfast, Belfast, Northern Ireland.

[5]Medical Faculty Heidelberg, Department of Anaesthesiology, Pain Medicine Section, Heidelberg University, Heidelberg, Germany.

[6]Department of General Internal Medicine and Psychosomatics, University Hospital Heidelberg, Heidelberg, Germany.

[7]Institute for General Practice and Interprofessional Care, Tübingen University Hospital, Tübingen, Germany.

[8]School of Pharmacy and Pharmaceutical Sciences, Trinity College Dublin, Republic of Ireland.

[9]Institute of Pharmacy, Freie Universität Berlin, Berlin, Germany.

[10]Primary Health Care, Leiden University Medical Center, Leiden, The Netherlands.

[11]Department of Clinical Pharmacy, Institute of Pharmacy, University of Bonn, Bonn, Germany.

*Corresponding author:

Heidelberg University, Medical Faculty Heidelberg / Heidelberg University Hospital, Medical Clinic, Internal Medicine IX – Department of Clinical Pharmacology and Pharmacoepidemiology, Cooperation Unit Clinical Pharmacy, Im Neuenheimer Feld 410, 69120, Heidelberg, Germany

Mail: hanna.seidling@med.uni-heidelberg.de

Supplementary file 3. Sociodemographic and general data of the experts.

Sociodemographic data of the expert panel (N =8).*

**Category**	**Number of answers (n)**
Professional background *(multiple answers possible)*	
Pharmacist	5
Physician	2
Researcher	6
Extent to which they consider their work practical or research-oriented	
Mostly practical	0
Balanced	4
Mostly research-oriented	4
Experience with… *(multiple answers possible)*	
conducting medication reviews	4
evaluating medication review studies	4
Expertise in an outcome included in the Core Outcome Set *(multiple answers possible)*	
Health-Related Quality of Life	5
Pain relief	2
Overuse	5
Underuse	5
Potentially inappropriate medications	7
Clinically significant drug-drug interactions	6
Drug-related hospitalization	5

**Answers were missing from 2 participants*
