# Supplementary material for: Development of a Core Outcome Measurement Set for studies of community pharmacist-led medication reviews in older patients with multimorbidity and polypharmacy
Source: Int J Clin Pharm. 2026 Sep 16;48(6):2467–78. doi: 10.1007/s11096-026-02223-y (PMC13731522; doi:10.1007/s11096-026-02223-y)
Supplement: Supplementary file 4 [file 11096_2026_2223_MOESM4_ESM.pdf]

Development of a Core Outcome Measurement Set for studies of community pharmacy-led medication reviews in older patients with multimorbidity and polypharmacy

Cathrin J. Vogt[1], Christiane Eickhoff[2], Veerle Foulon[3], Carmel Hughes[4], Jens Kessler[5], Cinara Paul[6,7], Cristín Ryan[8], Martin Schulz[2,9], Martina Teichert[10], Ronja Woltersdorf[11], Viktoria S. Wurmbach[1], Hanna M. Seidling[1]*

[1]Heidelberg University, Medical Faculty Heidelberg / Heidelberg University Hospital, Medical Clinic, Internal Medicine IX – Department of Clinical Pharmacology and Pharmacoepidemiology, Cooperation Unit Clinical Pharmacy, Im Neuenheimer Feld 410, 69120, Heidelberg, Germany.

[2]Department of Medicine, ABDA-Federal Union of German Associations of Pharmacists, Berlin, Germany.

[3]Department of Pharmaceutical and Pharmacological Sciences, KU Leuven, Belgium.

[4]School of Pharmacy, Queen's University Belfast, Belfast, Northern Ireland.

[5]Medical Faculty Heidelberg, Department of Anaesthesiology, Pain Medicine Section, Heidelberg University, Heidelberg, Germany.

[6]Department of General Internal Medicine and Psychosomatics, University Hospital Heidelberg, Heidelberg, Germany.

[7]Institute for General Practice and Interprofessional Care, Tübingen University Hospital, Tübingen, Germany.

[8]School of Pharmacy and Pharmaceutical Sciences, Trinity College Dublin, Republic of Ireland.

[9]Institute of Pharmacy, Freie Universität Berlin, Berlin, Germany.

[10]Primary Health Care, Leiden University Medical Center, Leiden, The Netherlands.

[11]Department of Clinical Pharmacy, Institute of Pharmacy, University of Bonn, Bonn, Germany.

*Corresponding author:

Heidelberg University, Medical Faculty Heidelberg / Heidelberg University Hospital, Medical Clinic, Internal Medicine IX – Department of Clinical Pharmacology and Pharmacoepidemiology, Cooperation Unit Clinical Pharmacy, Im Neuenheimer Feld 410, 69120, Heidelberg, Germany

Mail: hanna.seidling@med.uni-heidelberg.de

Supplementary file 4. PRISMA flow diagrams for the selection of OMIs for Health-related Quality of Life and pain relief.

First, a systematic search of published systematic reviews was conducted to identify existing outcome measurement instruments (OMIs) for Health-related Quality of Life and pain relief. In a subsequent step, it was searched for studies published after the search dates of the included reviews in order to capture newly developed or newly validated OMIs. The searches were conducted using a methodology broadly aligned with the PRISMA guideline. For example, a search strategy was developed and piloted (see Supplementary file 2) and subsequently applied to multiple databases (PubMed, Embase, COSMIN). Title/Abstracts and full texts were screened by two independent reviewers, by any discrepancies discussed and resolved by consensus. However, unlike a conventional systematic review no bias assessment was conducted. However, the primary objective was to identify relevant OMIs rather than to evaluate the quality of evidence or estimate intervention effects.

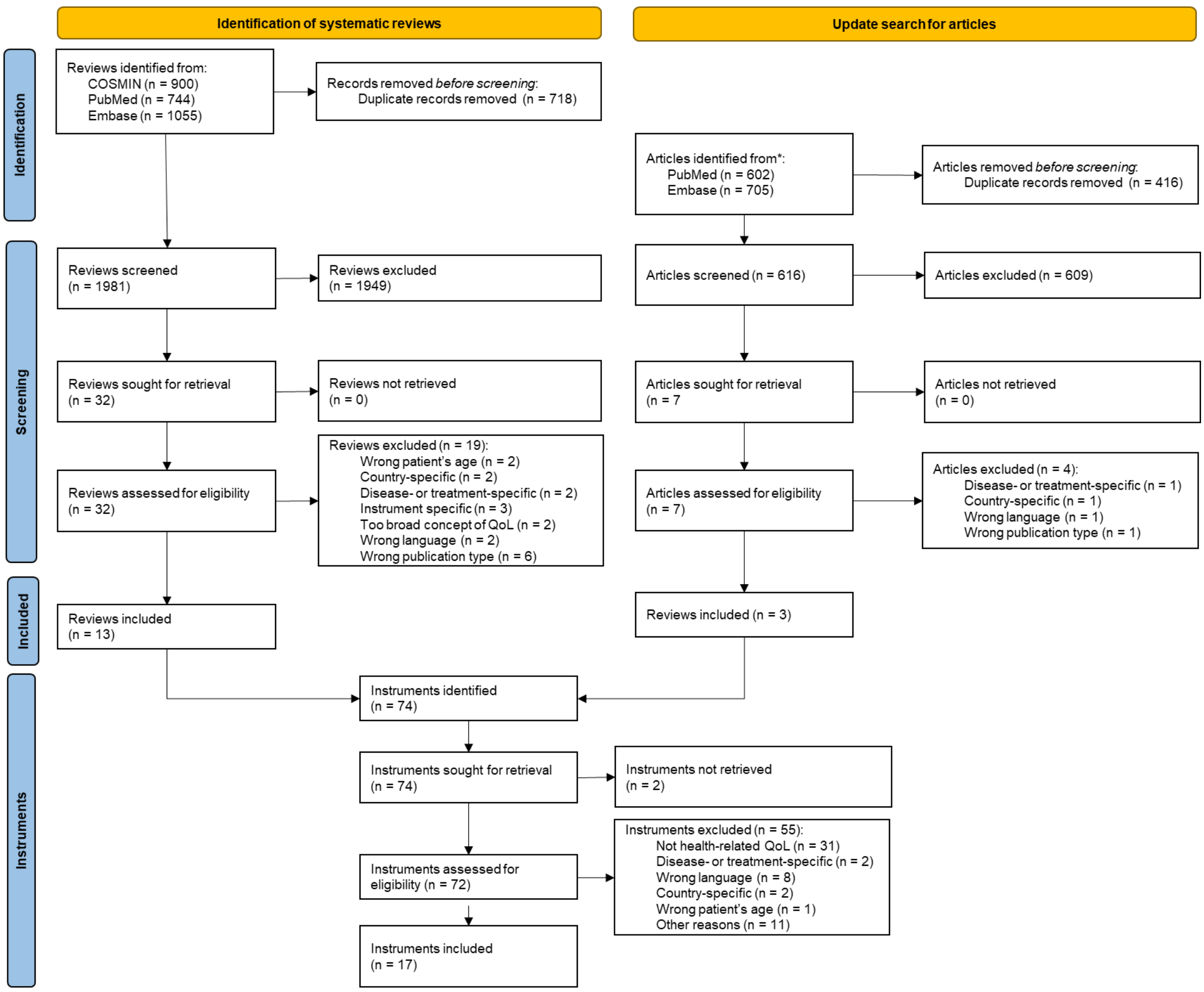

Figure S1: Prisma flow diagram for the search of health-related quality of life instruments.

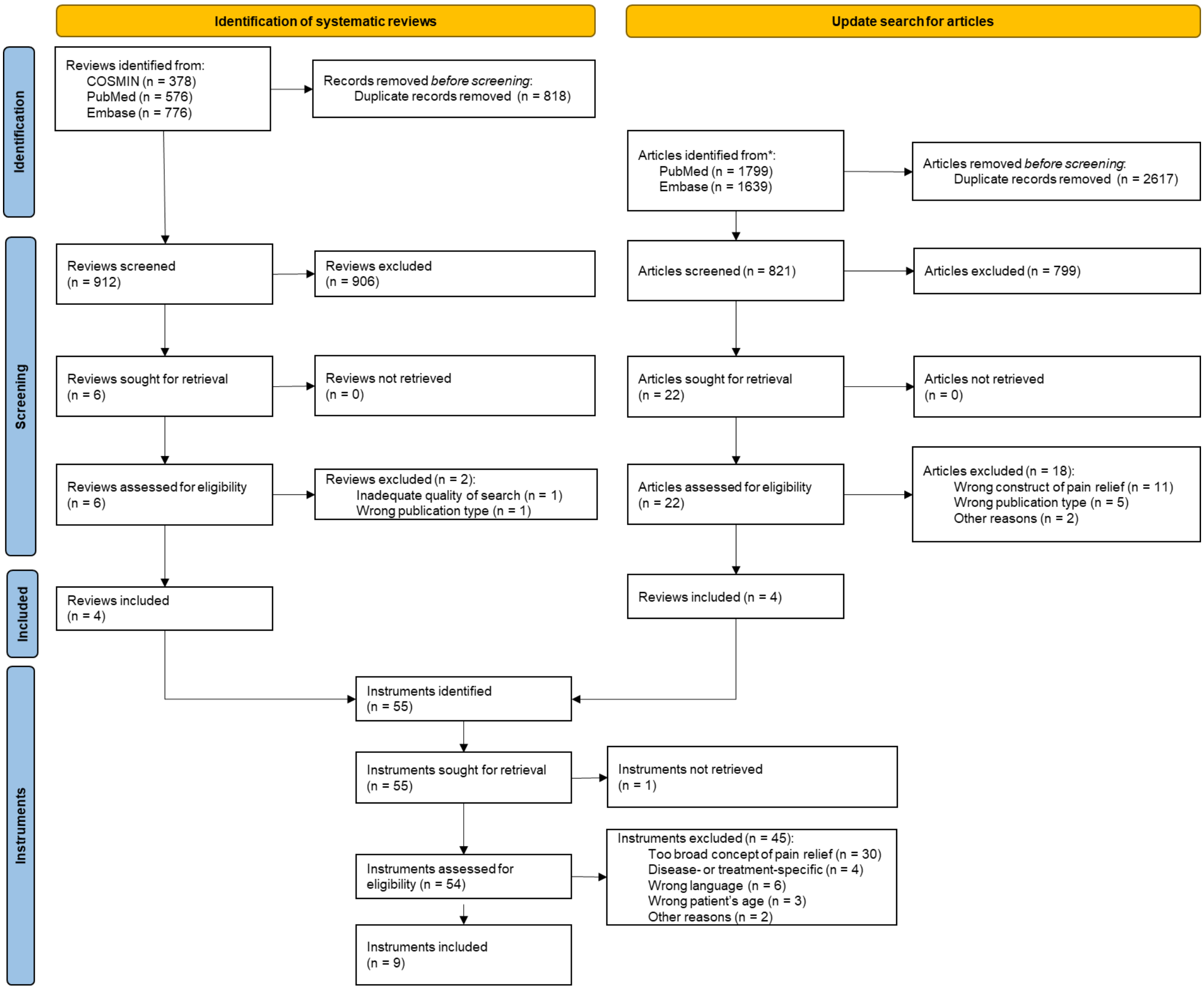

Figure S2: Prisma flow diagram for the search of pain relief instruments.
