# Supplementary material for: Development of a Core Outcome Measurement Set for studies of community pharmacist-led medication reviews in older patients with multimorbidity and polypharmacy
Source: Int J Clin Pharm. 2026 Sep 16;48(6):2467–78. doi: 10.1007/s11096-026-02223-y (PMC13731522; doi:10.1007/s11096-026-02223-y)
Supplement: Supplementary file 5 [file 11096_2026_2223_MOESM6_ESM.pdf]

Development of a Core Outcome Measurement Set for studies of community pharmacy-led medication reviews in older patients with multimorbidity and polypharmacy

Cathrin J. Vogt[1], Christiane Eickhoff[2], Veerle Foulon[3], Carmel Hughes[4], Jens Kessler[5], Cinara Paul[6,7], Cristín Ryan[8], Martin Schulz[2,9], Martina Teichert[10], Ronja Woltersdorf[11], Viktoria S. Wurmbach[1], Hanna M. Seidling[1]*

[1]Heidelberg University, Medical Faculty Heidelberg / Heidelberg University Hospital, Medical Clinic, Internal Medicine IX – Department of Clinical Pharmacology and Pharmacoepidemiology, Cooperation Unit Clinical Pharmacy, Im Neuenheimer Feld 410, 69120, Heidelberg, Germany.

[2]Department of Medicine, ABDA-Federal Union of German Associations of Pharmacists, Berlin, Germany.

[3]Department of Pharmaceutical and Pharmacological Sciences, KU Leuven, Belgium.

[4]School of Pharmacy, Queen's University Belfast, Belfast, Northern Ireland.

[5]Medical Faculty Heidelberg, Department of Anaesthesiology, Pain Medicine Section, Heidelberg University, Heidelberg, Germany.

[6]Department of General Internal Medicine and Psychosomatics, University Hospital Heidelberg, Heidelberg, Germany.

[7]Institute for General Practice and Interprofessional Care, Tübingen University Hospital, Tübingen, Germany.

[8]School of Pharmacy and Pharmaceutical Sciences, Trinity College Dublin, Republic of Ireland.

[9]Institute of Pharmacy, Freie Universität Berlin, Berlin, Germany.

[10]Primary Health Care, Leiden University Medical Center, Leiden, The Netherlands.

[11]Department of Clinical Pharmacy, Institute of Pharmacy, University of Bonn, Bonn, Germany.

*Corresponding author:

Heidelberg University, Medical Faculty Heidelberg / Heidelberg University Hospital, Medical Clinic, Internal Medicine IX – Department of Clinical Pharmacology and Pharmacoepidemiology, Cooperation Unit Clinical Pharmacy, Im Neuenheimer Feld 410, 69120, Heidelberg, Germany

Mail: hanna.seidling@med.uni-heidelberg.de

Supplementary file 6. Content-related quality criteria tested on a sample of instruments for overuse, underuse, and PIMs.

Recommended content-related quality criteria of appropriate outcome measurement instruments (OMIs) for overuse, underuse and/or PIMs piloted on a sample of OMIs. These quality criteria are based on the definition of the core outcomes of Beuscart et al. [1]. It is specified which criteria are most important for particular outcomes and outlining additional aspects to consider within each criterion.

	Content-related quality criteria						
	Stopping inappropriate medication	***Starting appropriate medication***	***Alternatives suggested***	***Dosing***	***Duration of therapy***	***Therapeutic duplications***	***Drug-disease interactions***
	Specification of quality criteria: linked core outcomes and additional considerations						
	Important for overuse	*Important for underuse*	*Important for PIMs*	*Including the impact of organ impairment (e.g. of the liver or kidneys) on dosing*	*Important for overuse and underuse*	*Important for overuse*	*Important for PIMs, not as important for overuse/underuse*
Australian Prescribing Indicators Tool [2]	✓	✓		✓	✓	✓	✓
Austrian Consensus List [3]	✓		✓				
Beers Criteria [4]	✓			✓	✓	✓	✓
EU (7)-PIM List [5],	✓		✓	✓	✓		
FORTA list [6]	✓	✓		✓			
French Consensus Panel List [7]	✓		✓	✓		✓	✓
GheOP3S [8]	✓		✓	✓	✓		✓
HEDIS [9]	✓	✓		✓			✓
IPET [10]	✓						✓
Italian criteria [11]	✓			✓	✓		
Korean criteria [12]	✓		✓	✓			✓
Lindblad criteria [13]	✓						✓
McLeod's criteria [14]	✓		✓				✓

New Mexico criteria [15]	✓		✓	✓			
NORGEP [16]	✓			✓			✓
Priscus List [17]	✓		✓	✓	✓		✓
Rancourt criteria [18]	✓			✓	✓	✓	
RASP 2.0 [19],	✓			✓	✓	✓	✓
Screening Tool for Older Person's Appropriate Prescriptions for Japanese [20]	✓	✓	✓	✓	✓		✓
START + STOPP criteria [21]	✓	✓		✓	✓	✓	✓
Taiwan criteria [22]	✓		✓	✓	✓		✓
Thailand criteria [23]	✓		✓	✓			✓
Zhan's criteria [24]	✓						

**GheOP3S = Ghent Older People's Prescriptions community Pharmacy Screening, HEDIS = Health Plan Employer Data and Information Set, IPET = Improving Prescribing in the Elderly Tool, NORGEP = Norwegian General Practice, PIM = Potentially inappropriate medication, RASP 2.0 = Rationalization of Home Medication by an Adjusted STOPP list in Older Patients, START = Screening Tool to Alert doctors to Right Treatment, STOPP =Screening Tool of Older Persons' potentially inappropriate Prescriptions.*

References

1. Beuscart JB, Knol W, Cullinan S, et al. International core outcome set for clinical trials of medication review in multi-morbid older patients with polypharmacy. *BMC Med.* 2018;16:21-30. DOI: 10.1186/s12916-018-1007-9.
2. Basger BJ, Chen TF, Moles RJ. Inappropriate medication use and prescribing indicators in elderly Australians: development of a prescribing indicators tool. *Drugs Aging.* 2008;25:777-793. DOI: 10.2165/00002512-200825090-00004.
3. Mann E, Böhmdorfer B, Frühwald T, et al. Potentially inappropriate medication in geriatric patients: the Austrian consensus panel list. *Wien Klin Wochenschr.* 2012;124:160-169. DOI: 10.1007/s00508-011-0061-5.
4. American Geriatrics Society 2023 updated AGS Beers Criteria® for potentially inappropriate medication use in older adults. *J Am Geriatr Soc.* 2023;71:2052-2081. DOI: 10.1111/jgs.18372.
5. Renom-Guiteras A, Meyer G, Thürmann PA. The EU(7)-PIM list: a list of potentially inappropriate medications for older people consented by experts from seven European countries. *Eur J Clin Pharmacol.* 2015;71:861-875. DOI: 10.1007/s00228-015-1860-9.
6. Pazan F, Weiss C, Wehling M. The FORTA (Fit fOR The Aged) List 2021: Fourth Version of a Validated Clinical Aid for Improved Pharmacotherapy in Older Adults. *Drugs Aging.* 2022;39:245-247. DOI: 10.1007/s40266-022-00922-5.
7. Laroche ML, Charmes JP, Merle L. Potentially inappropriate medications in the elderly: a French consensus panel list. *Eur J Clin Pharmacol.* 2007;63:725-731. DOI: 10.1007/s00228-007-0324-2.
8. Tommelein E, Petrovic M, Somers A, Mehuys E, van der Cammen T, Boussery K. Older patients' prescriptions screening in the community pharmacy: development of the Ghent Older People's Prescriptions community Pharmacy Screening (GheOP³S) tool. *J Public Health (Oxf).* 2016;38:e158-170. DOI: 10.1093/pubmed/fdv090.
9. Pugh MJ, Marcum ZA, Copeland LA, et al. The quality of quality measures: HEDIS® quality measures for medication management in the elderly and outcomes associated with new exposure. *Drugs Aging.* 2013;30:645-654. DOI: 10.1007/s40266-013-0086-8.
10. Naugler CT, Brymer C, Stolee P, Arcese ZA. Development and validation of an improving prescribing in the elderly tool. *Can J Clin Pharmacol.* 2000;7:103-107. DOI:
11. Maio V, Del Canale S, Abouzaid S. Using explicit criteria to evaluate the quality of prescribing in elderly Italian outpatients: a cohort study. *J Clin Pharm Ther.* 2010;35:219-229. DOI: 10.1111/j.1365-2710.2009.01094.x.
12. Kim DS, Heo SI, Lee SH. Development of a list of potentially inappropriate drugs for the korean elderly using the delphi method. *Healthc Inform Res.* 2010;16:231-252. DOI: 10.4258/hir.2010.16.4.231.
13. Lindblad CI, Hanlon JT, Gross CR, et al. Clinically important drug-disease interactions and their prevalence in older adults. *Clin Ther.* 2006;28:1133-1143. DOI: 10.1016/j.clinthera.2006.08.006.
14. McLeod PJ, Huang AR, Tamblyn RM, Gayton DC. Defining inappropriate practices in prescribing for elderly people: a national consensus panel. *Cmaj.* 1997;156:385-391. DOI:
15. Bachyrycz A, Dodd MA, Priloutskaya G. Development and dissemination of a statewide system to minimize use of Potentially Inappropriate Medications (PIMs). *Med Care.* 2012;50:993-996. DOI: 10.1097/MLR.0b013e31826ecfdc.
16. Rognstad S, Brekke M, Fetveit A, Spigset O, Wyller TB, Straand J. The Norwegian General Practice (NORGEP) criteria for assessing potentially inappropriate prescriptions to elderly patients. A modified Delphi study. *Scand J Prim Health Care.* 2009;27:153-159. DOI: 10.1080/02813430902992215.
17. Mann NK, Mathes T, Sönnichsen A, et al. Potentially Inadequate Medications in the Elderly: PRISCUS 2.0. *Dtsch Arztebl Int.* 2023;120:3-10. DOI: 10.3238/arztebl.m2022.0377.

18. Rancourt C, Moisan J, Baillargeon L, Verreault R, Laurin D, Grégoire JP. Potentially inappropriate prescriptions for older patients in long-term care. *BMC Geriatr.* 2004;4:9-18. DOI: 10.1186/1471-2318-4-9.

19. Van der Linden L. DL, Flamaing, Spriet, Willems, Milisen, Boonen, Tournoy. Development and validation of the RASP list (Rationalization of Home Medication by an Adjusted STOPP list in Older Patients): A novel tool in the management of geriatric polypharmacy. *Eur Geriatr Med.* 2014;5:175-180.

20. Kojima T, Mizukami K, Tomita N, et al. Screening Tool for Older Persons' Appropriate Prescriptions for Japanese: Report of the Japan Geriatrics Society Working Group on "Guidelines for medical treatment and its safety in the elderly". *Geriatr Gerontol Int.* 2016;16:983-1001. DOI: 10.1111/ggi.12890.

21. O'Mahony D, Cherubini A, Guiteras AR, et al. STOPP/START criteria for potentially inappropriate prescribing in older people: version 3. *Eur Geriatr Med.* 2023;14:625-632. DOI: 10.1007/s41999-023-00777-y.

22. Chang CB, Lai HY, Hwang SJ, et al. The updated PIM-Taiwan criteria: a list of potentially inappropriate medications in older people. *Ther Adv Chronic Dis.* 2019;10:2040622319879602. DOI: 10.1177/2040622319879602.

23. Winit-Watjana W, Sakulrat P, Kespichayawattana J. Criteria for high-risk medication use in Thai older patients. *Arch Gerontol Geriatr.* 2008;47:35-51. DOI: 10.1016/j.archger.2007.06.006.

24. Zhan C, Sangl J, Bierman AS, et al. Potentially inappropriate medication use in the community-dwelling elderly: findings from the 1996 Medical Expenditure Panel Survey. *Jama.* 2001;286:2823-2829. DOI: 10.1001/jama.286.22.2823.
